# Nrf2: A key regulator in chemoradiotherapy resistance of osteosarcoma

**DOI:** 10.1016/j.gendis.2024.101335

**Published:** 2024-05-22

**Authors:** Xianglin Peng, Jing Feng, Han Yang, Ping Xia, Feifei Pu

**Affiliations:** a Department of Orthopedics, Wuhan Hospital of Traditional Chinese and Western Medicine, Tongji Medical College, Huazhong University of Science and Technology, Wuhan 430022, China; b Department of Orthopedics, Wuhan No.1 Hospital, Wuhan 430022, China; c Special Key Laboratory of Gene Detection and Therapy of Guizhou Province, Zunyi Medical University, Zunyi 563000, China; d Department of Immunology, Zunyi Medical University, Zunyi 563000, China; e Department of Orthopedics, Wuhan Fourth Hospital, Wuhan 430030, China

**Keywords:** Carcinogenesis, Nrf2, Osteosarcoma, Resistance, Therapeutic strategies

## Abstract

Osteosarcoma (OS), frequently observed in children and adolescents, is one of the most common primary malignant tumors of the bone known to be associated with a high capacity for invasion and metastasis. The incidence of osteosarcoma in children and adolescents is growing annually, although improvements in survival remain limited. With the clinical application of neoadjuvant chemotherapy, chemotherapy combined with limb-preserving surgery has gained momentum as a major intervention. However, certain patients with OS experience treatment failure owing to chemoradiotherapy resistance or metastasis. Nuclear factor E2-related factor 2 (Nrf2), a key antioxidant factor in organisms, plays a crucial role in maintaining cellular physiological homeostasis; however, its overactivation in cancer cells restricts reactive oxygen species production, promotes DNA repair and drug efflux, and ultimately leads to chemoradiotherapy resistance. Recent studies have also identified the functions of Nrf2 beyond its antioxidative function, including the promotion of proliferation, metastasis, and regulation of metabolism. The current review describes the multiple mechanisms of chemoradiotherapy resistance in OS and the substantial role of Nrf2 in the signaling regulatory network to elucidate the function of Nrf2 in promoting OS chemoradiotherapy resistance and formulating relevant therapeutic strategies.

## Introduction

Osteosarcoma (OS), the most common primary malignant bone tumor in children and adolescents, occurs predominantly in the metaphysis of the tubular bone and is characterized by a tendency to metastasize.^1^ Before 1970, the management of OS was limited to amputation and adjuvant radiotherapy; however, patients who underwent surgery presented a persistently high mortality rate and a five-year survival rate of ∼20%.^2^^,^^3^ Subsequent adjuvant chemotherapy is employed to reduce the tumor size preoperatively for limb-preserving surgery and eradicate small metastatic lesions. Neoadjuvant chemotherapy includes cisplatin (DDP), doxorubicin (DOX), methotrexate (MTX), and ifosfamide (IFO).^4^ Although chemotherapy has successfully improved the five-year survival rate of patients with non-metastatic OS to 70%,^5^^,^^6^ radiotherapy and chemotherapy failed to improve survival in ∼20% of patients with OS and extraosseous metastases.^7^ The effect of chemoradiotherapy is markedly limited in OS found to be resistant prior to therapy,^8^ and OS cell resistance has been implicated in chemotherapy failure observed in 90% of patients.^9^ Chemotherapy or radiotherapy alone can be ineffective in treating patients with OS, while radiotherapy remains controversial owing to its poor tolerability and high incidence of wound complications.^10^ Currently, surgical resection is the main therapy for OS, but patients with extensive metastases still relapse. Growing evidence suggests that chemotherapy combined with surgical resection can effectively clear primary lesions and small metastases, improving patient survival.^11^ However, the rapid emergence of resistant cells complicates the clinical treatment of OS, with certain patients failing to respond to chemotherapy owing to drug resistance, ultimately resulting in tumor relapse or distant metastasis and death. Therefore, drug resistance also critically impacts the treatment success of patients with OS, and it is imperative to further elucidate the mechanism of OS chemoradiotherapy resistance and explore therapeutic regimens capable of reversing chemoradiotherapy resistance.^12^

Regulatory mechanisms underlying OS resistance are complex and related to drug efflux, cellular detoxification, DNA damage repair, and regulated cell death (RCD), with crosstalk between the different mechanisms regulating each other.^9^ Despite extensive research on OS resistance-related proteins, DNA damage repair, programmed RCD, and noncoding RNAs, emerging areas, such as ferroptosis, pyroptosis, and RCD crosstalk, remain poorly explored.^13^ Multiple chemotherapeutic agents can reportedly promote RCD by increasing cellular oxidative stress, and an imbalance in redox homeostasis is key to chemotherapy resistance, accompanied by increased levels of antioxidant proteins and inhibition of reactive oxygen species (ROS) generation in resistant OS cells. Ferroptosis is a type of RCD dependent on lipid peroxidation, closely related to oxidative stress, tumor resistance, and crosstalk with autophagy.^14^ The excessive antioxidative stress capacity of drug-resistant cancer cells additionally mediates radiotherapy resistance, especially in cancers such as OS, which are insensitive to radiotherapy. Therefore, reducing the cellular antioxidant stress capacity is crucial for reversing chemoradiotherapy resistance in OS.

Nuclear factor E2-related factor 2 (Nrf2) is a key transcription factor that activates the antioxidant response element (ARE), combining with ARE to initiate the transcription and translation of downstream genes, such as phase II detoxification enzymes, antioxidant proteins, and proteasomes, to mobilize the antioxidant stress enzyme system and prevent tissues from excessive ROS damage.^15^ Nrf2 and these target genes play substantial roles in OS progression and drug resistance by regulating drug metabolism, efflux, energy metabolism, amino acid metabolism, DNA repair, mitochondrial damage, proliferation, metastasis, autophagy, ferroptosis, and apoptosis.^16^ Nrf2 was initially considered a tumor suppressor gene owing to its function in maintaining cellular physiological homeostasis and preventing endogenous and exogenous damage, with studies in mice revealing that Nrf2 deficiency increases susceptibility to cancer.^17^ Conversely, Nrf2 expression was found to be substantially elevated in OS cells and negatively correlated with the five-year survival of patients.^18^ Moreover, Nrf2 hyperactivation provides a favorable environment for tumor cell survival, including protection from oxidative stress, reduction in intracellular chemotherapeutic drug concentrations, and mediation of chemoradiotherapy resistance.^19^ Thus, Nrf2 might be an oncogene in OS, promoting OS progression and resistance via multiple pathways. The development of Nrf2 inhibitors as new anti-OS therapies affords a promising option. However, the clinical application of Nrf2 remains limited; only a few broccoli extracts have entered clinical trials, demonstrating unsatisfactory results. Therefore, we reviewed the principal mechanisms underlying chemoradiotherapy resistance in OS, the protein structure of the Kelch-like ECH-associated protein 1 (Keap1)/Nrf2/ARE pathway, and its regulatory mechanisms. Moreover, we detailed the critical role of Nrf2 in the progression of OS and chemoradiotherapy resistance, along with recent advances in Nrf2-based clinical trials and preclinical experiments, to furnish novel concepts for developing new anti-OS therapeutic strategies.

## Chemoradiotherapy resistance mechanisms and countermeasures of OS

**Figure 1:**
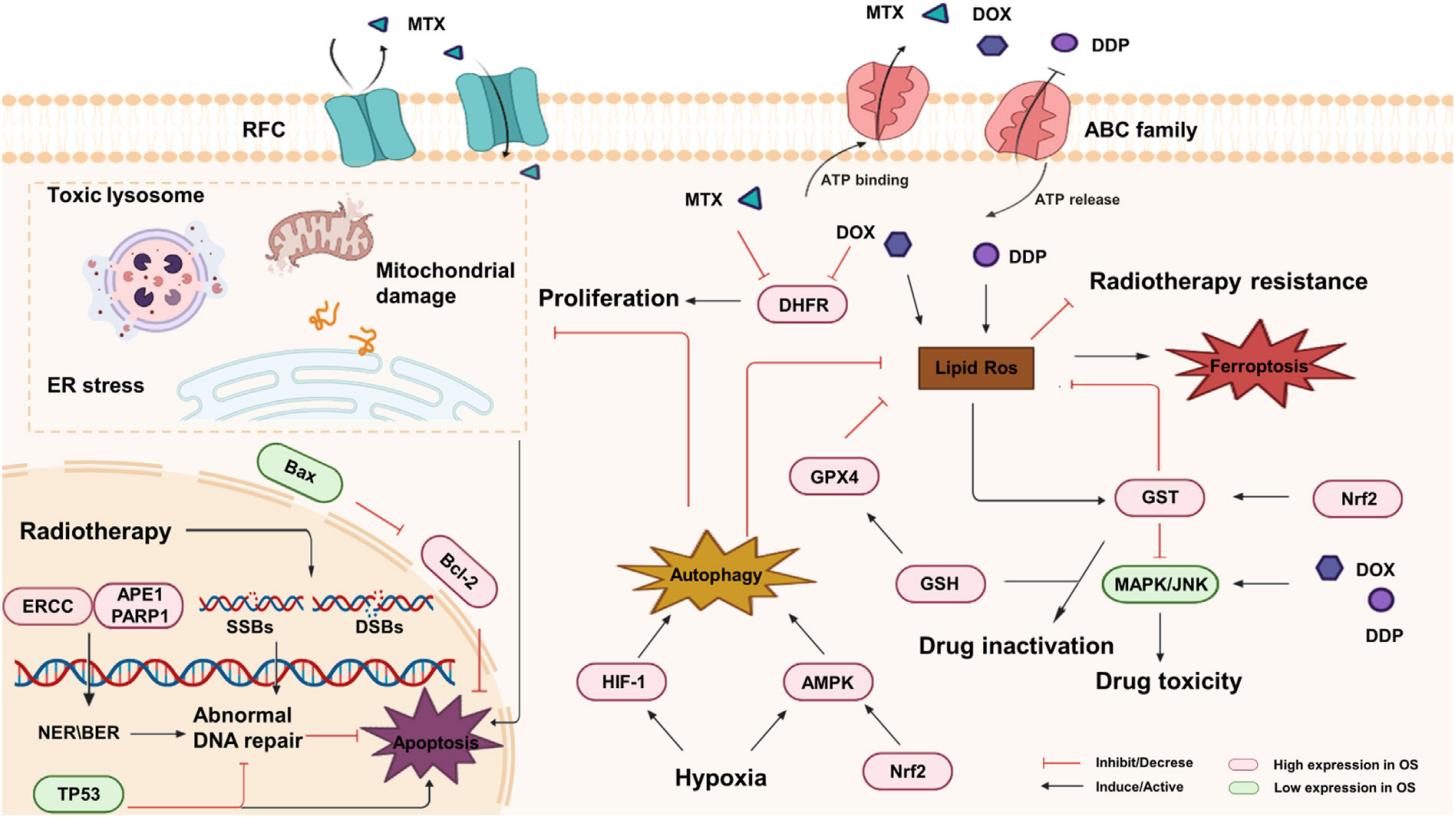
Mechanisms of resistance to chemoradiotherapy in osteosarcoma. In chemoradiotherapy resistant OS cells, altered expression of the drug transporter proteins, RFC and ABC family, reduces intracellular drug concentrations. The tumor microenvironment and changes in signaling pathways allow OS cells to escape RCD. Abnormal expression of DNA repair-related proteins promotes DNA repair and allows OS cells to avoid DNA damage leading to RCD. RCD, regulated cell death.

In cancer cells, drug resistance is categorized as intrinsic and acquired. While almost half of the cancers show resistance before treatment, i.e., intrinsic resistance, 50% of remaining cancers develop resistance to treatment, i.e., acquired resistance.^20^ Different chemotherapeutic agents possess distinct mechanisms of action; consequently, cancer cells develop resistance via distinct mechanisms, including oncogene mutations, altered expression of signaling pathways, and changes in the tumor microenvironment.^21^^,^^22^ These changes lead to abnormal pathological or physiological phenomena in cancer cells, summarized as follows: (1) reduced intracellular drug concentration, (2) RCD regulation, and (3) repair of abnormal DNA (Fig. 1). Multiple Nrf2 target genes are known to promote OS resistance by regulating these resistance-related pathophysiological phenomena. Thus, in this section, we detail the mechanisms of chemoradiotherapy resistance.

### Reduction in intracellular drug concentration

Increased drug efflux is a substantial contributor to OS resistance, primarily mediated via the enhanced expression of the ATP-binding cassette (ABC) family in resistant cancer cells. The ABC family is an ATP-dependent membrane transport protein that pumps multiple drugs out of cells via ATP hydrolysis, which supplies energy. The ABC family primarily comprises seven families: P-glycoprotein (P-gp/ABCB1), multidrug resistance protein 2 (MRP2/ABCC2), and breast cancer resistance protein (BCRP/ABCG2).^23^ Typically, cancer cells exposed to a single chemotherapy drug develop acquired resistance, exhibiting resistance to multiple other chemotherapeutic drugs, a phenomenon known as multidrug resistance (MDR). The overexpression of ABC family members is one of the key mechanisms of MDR in cancer cells.^24^ P-gp, the first MDR protein discovered, is also known as multidrug resistance protein 1 (MDR1). MDR1 can transport multiple chemotherapeutic drugs, such as DOX and DDP, extracellularly, decreasing intracellular drug concentrations and cytotoxicity.^25^ MDR1 expression levels were higher in DDP-resistant OS cells than in DDP-sensitive OS cells,^26^ and OS patients with high MDR1 expression reportedly exhibit a more than tripled risk of postoperative recurrence, adverse events,^27^ and a high mortality rate.^28^ In human OS cell lines, MDR1 expression increased with increasing DOX doses and exhibited cross-resistance to vincristine.^29^ Conversely, treatment of OS cells with radiotracers reduced tracer concentration and enhanced efflux in OS cells with high MDR1 expression.^30^

Other members of the ABC family have also been reported to mediate drug efflux in OS resistance, including ABCC1, found to be the second most drug-transporting protein related to OS resistance. The addition of ABCC1 and MDR1 inhibitors reversed the DOX-mediated sensitization of OS in drug-resistant OS cell lines.^31^ ABCC4 has also been associated with DOX resistance in OS cells,^32^ while ABCC5 was found to be overexpressed in patients with poor prognosis.^33^ Conversely, OS mice with decreased ABCG2 expression showed better DOX efficacy.^34^ Reportedly, modulating the expression of ABC family proteins could reverse OS resistance to chemotherapeutic drugs. Daclatinib, a novel tyrosine kinase inhibitor (TKI) and an inhibitor of ABC transporter proteins, reportedly reversed drug resistance in OS cells by antagonizing MDR1- and ABCG2-mediated drug efflux.^35^ Similarly, anlotinib, a novel TKI, reversed DOX resistance in OS cells by suppressing MDR1, suggesting that combining TKI and DOX may be an effective treatment option for OS.^36^

Furthermore, decreased expression of reduced folate carrier (RFC) in OS has been shown to contribute to OS resistance to MTX; RFC, localized at the membrane, is the primary transporter protein for cellular intake of MTX.^37^ Poor efficacy of MTX chemotherapy was observed in OS cells with low RFC expression.^38^ Dihydrofolate reductase (DHFR), a target protein of MTX, is essential for cell proliferation and nucleic acid precursor synthesis.^39^ MTX-treated patients with OS tend to present high DHFR expression, accompanied by an increased probability of metastasis.^40^ Notably, primary OS tissues exhibit lower expression of both DHFR and RFC than metastatic OS tissues, suggesting that resistance to MTX in OS was acquired during treatment.^41^

### Regulation of cell death

Physiologically, RCD maintains homeostasis by eliminating damaged cells and organelles from the internal environment. RCD is a notable component of innate immunity under pathological conditions and during cancer progression.^42^ Cancer cells commonly exhibit increased metabolic levels, given the need for an adequate nutrient supply for proliferation, which results from the reprogramming of energy metabolism in cancer cells, and this alteration simultaneously exerts a long-term effect on cell fate. For example, redox reaction-generated ROS easily accumulated in cancer cells at high metabolic levels, thereby enhancing susceptibility to ferroptosis.^43^ RCD is an essential pathway for the antitumor effects of multiple chemotherapy drugs. However, it should be noted that cancer cells have evolved various mechanisms to evade RCD, which results in drug resistance.^44^ Elucidating the crosstalk mechanism between Nrf2 and RCD is necessary to overcome OS chemoradiotherapy resistance.

#### Ferroptosis and oxidative stress

Ferroptosis is an iron-dependent RCD driven by lipid peroxidation and modulated by multiple routes.^45^ Since its discovery in 2012, the substantial role of ferroptosis has been well-documented in cancer resistance, ischemic diseases, and neurodegenerative diseases.^46^ Multiple chemotherapy drugs, including DDP and DOX, can induce ROS generation and accumulation, thereby promoting ferroptosis, exacerbating the ROS load of cancer cells on the one hand while conversely inducing adverse effects, such as acute kidney injury and myocardial toxicity.^47^^,^^48^ OS cells can escape ferroptosis by modulating the expression of various ferroptosis-related proteins, which also leads to reduced drug sensitivity. Increasing the sensitivity of OS cells to ferroptosis can be valuable in decreasing the drug dosage, thereby mitigating side effects. Elevated Nrf2 and glutathione peroxidase 4 (GPX4) expression was documented in OS cells that developed resistance after DDP treatment, and the combined application of erastin, a ferroptosis agonist, substantially reversed OS cell resistance.^49^ GPX4, a predominant rate-limiting enzyme in ferroptosis, neutralizes intracellular phospholipid hydroperoxides (PLOOHs), a form of ROS, and erastin can induce ferroptosis by inhibiting GPX4.^50^

DDP-resistant OS cells reportedly exhibit increased expression of antioxidants glutathione (GSH) and glutathione-S-transferase (GST), and patients with high GSH and GST expression had a high risk of OS and poor clinical prognosis.^51^^,^^52^ As a cofactor of GPX4, GSH is essential for protecting cells from various oxidative stress injuries; however, cancer cells utilize this mechanism to avoid ferroptosis.^53^ Conversely, inhibiting GSH expression in mice was found to induce ferroptosis, leading to antitumor effects.^54^ GST acts as a phase II detoxification enzyme that catalyzes the coupling of various drugs as substrates with GSH, leading to their inactivation. Notably, resistance to DOX and DDP arises indirectly owing to the GST-mediated suppression of the mitogen-activated protein kinase (MAPK), c-Jun N-terminal kinase (JNK) pathway rather than direct inactivation as a GST substrate.^55^ Various chemotherapeutic agents, including DDP and DOX, induce cytotoxicity by activating the AMPK-JNK pathway. GST can also downregulate ROS to alleviate oxidative stress, while GST expression can be regulated by ROS levels, a potential adaptive response; however, abnormally high expression in OS cells is considered an important mechanism of DDP resistance.^56^ Nrf2 is an upstream stimulator of GST, highly expressed in OS cells, mediating resistance. Therefore, Nrf2 inhibition can effectively reverse drug resistance in OS cells.^57^

#### Autophagy and hypoxia

Autophagy is a physiological process widely observed in human cells and plays an important role in providing intracellular energy, maintaining intracellular homeostasis, and removing worthless cells. Under stressful conditions such as hypoxia, autophagosomes can catabolize damaged intracellular organelles and proteins to recycle intracellular material and provide energy to the cell.^58^ Autophagy is an innate immune defense mechanism against tumors and other pathogens. Although autophagy typically plays an active role in infectious diseases, it is often considered to promote cancer cell survival, given that autophagy removes damaged organelles and excess ROS, avoiding RCD.^59^ This homeostasis maintenance function in the intracellular environment is similar to Nrf2. In the context of oxidative stress, Nrf2 can also interact with autophagy to promote each other in a feedforward manner. Multiple chemotherapy drugs can induce autophagy in OS cells, and an increased level of autophagy promotes OS cell resistance to DOX, DDP, and MTX.^60^^,^^61^ Autophagy is reportedly regulated by cellular environments such as hypoxia, starvation, and cytotoxicity, or signaling pathways such as AMPK/mTORC1, PI3K/AKT/mTORC1, and Ras/Raf/MEK/ERK.^62^ The OS tumor microenvironment is characterized by hypoxia, which mediates OS resistance.^63^ Hypoxia-inducible factor-1 (HIF-1) is a transcription factor dependent on hypoxia. In addition to directly contributing to OS resistance by inducing MDR1 expression and decreasing ROS levels, HIF-1 can upregulate the expression of autophagy-related proteins (ATGs) and LC3 to promote autophagy at multiple stages of autophagosome elongation and maturation, thereby contributing to OS resistance.^64^ Poor sensitivity to radiotherapy is a characteristic of OS, with several studies demonstrating that HIF-1 can mediate radiotherapy resistance in OS by promoting autophagy to remove ROS.^65^^,^^66^ Inhibition of HIF-1 expression in a DOX-resistant OS cell line reversed the observed resistance.^67^ However, inhibiting HIF-1 expression in a hypoxic environment may be insufficient to reverse OS resistance, suggesting the crucial role of HIF-1-independent resistance.^68^ Hypoxia also induces AMPK signaling to mediate OS resistance to DOX, and this resistance is reversed upon AMPK inhibition.^69^

#### Apoptosis and cell cycle

Apoptosis, also known as programmed cell death, is an immune response involved in the clearance of senescent or damaged cells under physiological or pathological conditions. Reduced apoptosis is closely associated with tumor malignancy, metastasis, and drug resistance.^70^ This is mainly attributed to the abnormal expression of apoptosis-related proteins and signaling pathways in cancer cells, resulting in cell cycle arrest or cell proliferation, which is directed toward apoptosis.^71^^,^^72^ Apoptosis is an essential mechanism mediating the antitumor effects of various chemotherapeutic drugs, and the apoptotic signaling pathway is blocked in OS-resistant cells.^73^

In a genetic analysis of 1244 OS tissues, P53 (TP53), the most well-known oncogene, was identified as the most frequent pathogenic mutation (4.4%). In these TP53-mutated patients with OS, OS cells were more prone to occur in the midshaft bone and metastasize, and the survival rate was notably poorer than that in patients with OS without the mutation.^74^ TP53 deletion or mutation has been suggested to substantially mediate OS chemotherapy resistance.^75^ Mechanistically, TP53 exerts antitumor effects or promotes genotoxicity by inducing cell cycle arrest, apoptosis, and transcription of metabolism-related genes following DNA damage.^76^ OS cells transfected with R175H, a TP53 mutant, exhibited resistance to DDP, DOX, and radiotherapy, whereas suppressing R175H restored DOX-induced apoptosis and reversed drug resistance in OS cells.^77^ Although the cell cycle was retarded, drug-induced P53 expression did not induce apoptosis in OS cells; however, apoptosis was markedly induced when combined with DDP treatment to promote drug toxicity.^78^ Theaflavin, a component of tea, induced DNA damage and led to apoptosis in OS cells via TP53-dependent pathways without inducing in normal tissues. Therefore, the discovery of antitumor effects through dietary therapy may be crucial for OS prevention.^79^ Apoptosis also considerably contributes to radiotherapy efficacy, given that the most prominent effect of radiotherapy is ionizing radiation-mediated apoptosis induction, leading to DNA single-strand breaks (SSBs) and double-strand breaks (DSBs).^80^ TP53 mutations also mediate OS resistance to radiotherapy, and upregulated TP53 expression in combination with radiotherapy can enhance OS cell sensitivity to radiotherapy by inducing apoptosis in a TP53-dependent manner.^81^^,^^82^

The Bcl-2 family is widely known as the predominant apoptosis regulator and contains the antiapoptotic proteins Bcl-2 and Bcl-xL, and the proapoptotic protein Bax.^83^ Bax can form a heterodimer with Bcl-2 and counteract the antiapoptotic effects of Bcl-2; aberrant expression of both proteins can mediate OS resistance by affecting apoptosis.^84^ Various chemotherapeutic drugs exert antitumor effects by inducing OS cell apoptosis by downregulating Bcl-2 expression.^85^ The expression of Bcl-2 and Bcl-xL was substantially upregulated in DOX-resistant OS cells; this resistance was reversed by the simultaneous inhibition of Bcl-2 and Bcl-xL expression.^86^ Long noncoding RNA FOXD2-AS1 markedly reversed OS resistance to DDP by upregulating Bax and downregulating Blc-2 expression.^87^ Nrf2 can block apoptotic signaling by regulating the expression of Bcl-2 and Bax, thereby promoting OS resistance.

### Abnormal DNA repair

Cells are exposed to endogenous or exogenous DNA damage under physiological conditions, which, in addition to inducing apoptosis, may lead to cell cycle arrest and recruitment of DNA repair proteins for DNA repair, thereby preventing apoptosis.^79^ This includes several repair mechanisms such as direct reversal repair, base excision repair (BER), nucleotide excision repair (NER), homologous recombination repair, non-homologous end joining (NHEJ), and mismatch repair (MMR).^88^ Multiple chemotherapeutic drugs and radiotherapy reportedly induce RCD via DNA damage, and abnormal DNA repair mediates OS cell resistance.^89^

NER and BER are the most prominent mechanisms that mediate OS resistance, frequently accompanied by increased expression of excision repair cross-complementing (ERCC), glutathione S-transferase P1 (GSTP1), and poly ADP-ribose polymerase (PARP1).^90^ ERCC, a member of the NER family, was found to be positive in 26% of patients with OS and was markedly associated with event-free and overall survival.^91^ The ERCCI rs11615 polymorphism reportedly plays an essential role in OS in terms of chemosensitivity and overall survival in NER gene polymorphism studies.^92^^,^^93^ ERCC2 rs1799793 and rs13181 (A allele and GG genotype) are also associated with overall survival, suggesting the involvement of ERCC and NER in OS progression.^94^^,^^95^ Interference with the expression of ERCC1, ERCC2, ERCC3, and ERCC4 in DDP-resistant OS cell lines enhanced OS cell sensitivity to DDP. Two ERCC-targeting medications, NSC130813 (targeting the interaction between ERCC1 and ERCC4) and triptolide (targeting ERDC3), increased sensitivity to DDP by more than two-fold in both OS-resistant and parental cell lines.^89^

PARP1, a member of the BER family, recruits downstream molecules to repair DNA upon the onset of SSBs or DSBs, thereby preventing apoptosis.^96^ In patients with OS, PARP1 expression has been associated with poor survival, and PARP1 knockdown in OS cells or treatment with olaparib, a PARP1 inhibitor, substantially suppressed cell proliferation and induced apoptosis, eliciting better efficacy both *in vivo* and *ex vivo* when combined with DOX therapy.^97^ Preclinically, olaparib promoted DNA damage, G2/M phase blockade, apoptosis, and enhanced antitumor effects when combined with trabectedin.^98^ Apurinic endonuclease 1 (APE1), another key enzyme in BER, is highly expressed in 72% of OS tissues. siRNA-mediated reduced APE1 expression could enhance sensitivity to chemotherapy when compared with radiotherapy.^99^ OS cell lines with high APE1 expression exhibited a lower incidence of DDP-induced DNA damage and apoptosis than OS cell lines with low APE1 expression.^100^ Reportedly, mir-765 and APE1 are closely correlated with the clinical prognosis of patients with OS, and mir-765 can target APE1 and increase OS sensitivity to DDP.^101^ Moreover, mir-765 downregulated APE1 and angiogenic factors and, in combination with DDP treatment, reduced OS cell migration and angiogenesis *in vivo* and *in vitro*.^102^ In an OS graft tumor model, siRNA-mediated APE1 suppression, combined with endothelial inhibitor treatment, elicited a more pronounced increase in apoptosis and a decrease in vascular density.^103^ In two experiments using APE1 inhibitors combined with chemotherapy drugs, both APE1 inhibitor groups showed increased DNA damage and antitumor effects.^104^^,^^105^ GSTP1 is also a member of the BER, and elevated GSTP1 expression was found to be associated with OS recurrence and poor clinical prognosis.^106^ A GSTP1 inhibitor afforded good antitumor activity in DDP, ROX, and MTX-resistant OS cell lines, and combining a GSTP1 inhibitor with DDP may be a novel therapy in OS-resistant patients.^51^

## The Keap1/Nrf2/ARE system

**Figure 2:**
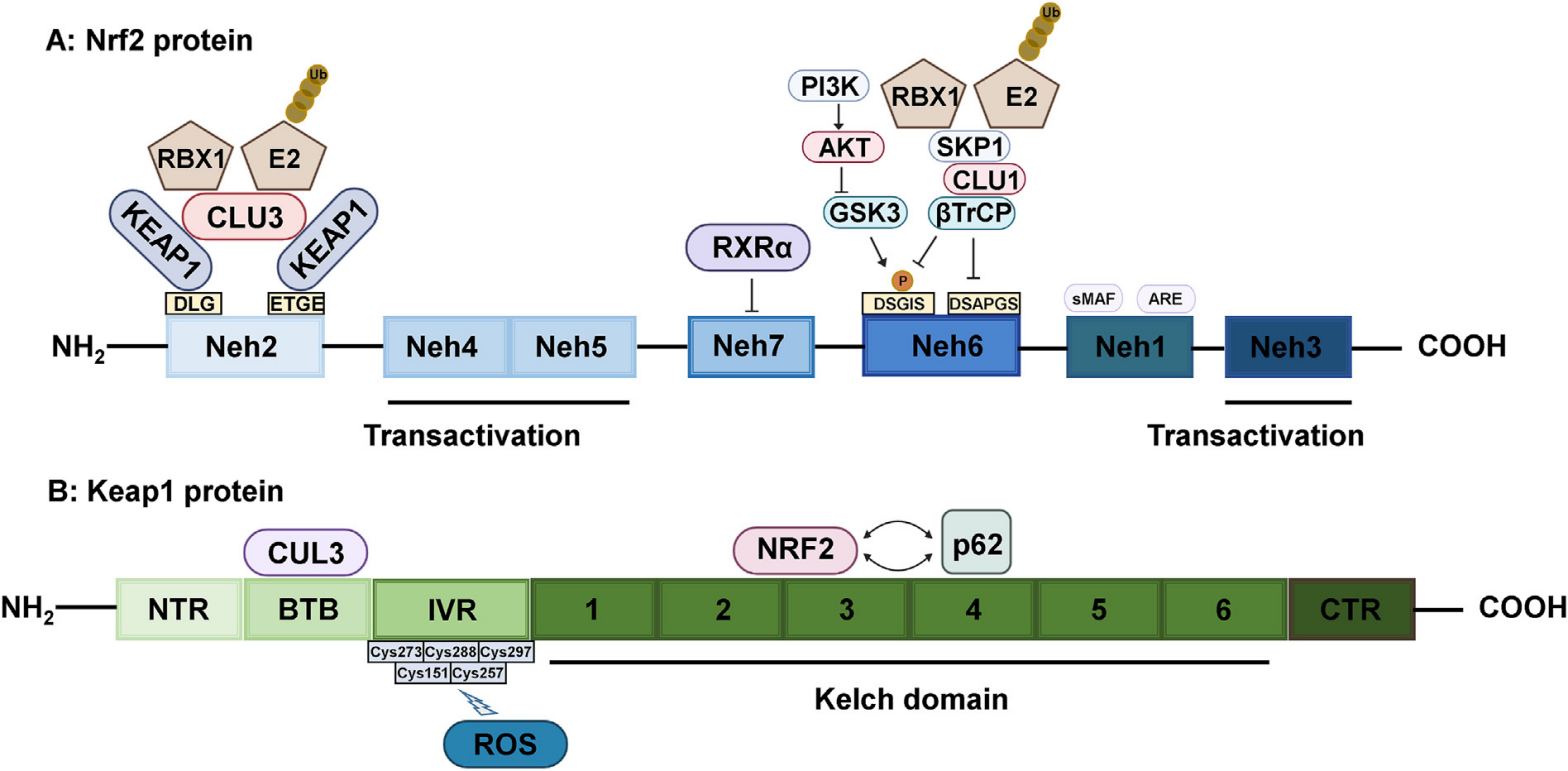
Nrf2 and Keap1 protein structures. A: The Nrf2 protein contains seven highly conserved Nrf2-ECH homologs (Neh), i.e., Neh1-Neh7, each exhibiting a distinct function. B: Keap1 is a major regulator of Nrf2 and comprises five structural domains: DGR (comprising six Kelch fragments), IVR, BTB, CTR, and NTR.

During oxidative stress, Keap1/Nrf2/ARE maintains intracellular redox homeostasis by inducing antioxidant gene expression.^107^ The Nrf2 protein contains seven highly conserved Nrf2-ECH homologs (Neh), i.e., Neh1-Neh7, each exhibiting a distinct function (Fig. 2A). Neh1, 3, 4, and 5 participate in downstream gene activation, and Neh1 can interact with the small MAF protein (sMAF) and bind to ARE to activate downstream target genes.^108^ Neh3, 4, and 5 are transactivating structural domains that bind to and interact with cotranscription factors.^109^ Nrf2 can be degraded by ubiquitination or by the 26S protease pathway, which is mainly dependent on Neh2 and Neh6. Neh2 contains two amino acid sequences (DLG and ETGE) recognized and bound by Keap1, and physiologically, Keap1 can bind to Nrf2 and induce its degradation.^110^ Neh6 is a serine-rich structural domain containing DSGIS and DSAPGS binding motifs, recognized by β-transducin repeat-containing protein (β-TrCP), where DGGIS recognition is dependent on GSK3 phosphorylation of serine residues. β-TrCP binds and induces Nrf2 degradation as a substrate receptor for the SKP1-CUL1-RBX1 ubiquitin ligase complex.^111^ GSK3, as a substrate for AKT, is also regulated by the PI3K/AKT pathway, and PI3K can substantially induce nuclear translocation of Nrf2 and attenuate ferroptosis.^112^ RXRα can bind Neh7 to inhibit the transcriptional activity of Nrf2.^113^

Keap1 is a major regulator of Nrf2 and comprises five structural domains: DGR (comprising six Kelch fragments), IVR, BTB, CTR, and NTR (Fig. 2B). DGR specifically binds to Nrf2. The BTB region is essential for Keap1 homodimerization and binding to the CUL3-E3 ubiquitin ligase complex. IVR is a cysteine-rich region involved in the functional regulation of Keap1 in response to oxidative stress.^114^ Under physiological conditions, DGR specifically binds to the Neh2 structural domain of Nrf2 and continuously degrades Nrf2 via the Cul3-Keap1 ubiquitin ligase complex, resulting in low Nrf2 levels.^115^ However, under oxidative stress, the highly reactive cysteine residues in the IVR region are oxidized, resulting in the dissociation of the low-affinity DLG motif of Nrf2 from the DGR region, whereas the high-affinity ETGE remains bound to DGR, preventing the recognition and degradation of Nrf2 by ubiquitylated ligases. Thus, Nrf2 can accumulate and translocate to the nucleus, recognizing ARE through its Neh1 structural domain, which, in turn, induces the activation of a range of antioxidant genes.^116^ The selective autophagy receptor p62 can competitively bind to the DGR region of Keap1, subsequently deregulating Keap1-mediated Nrf2 degradation by competing with Nrf2. The p62 enhancer contains ARE sequences that can be upregulated by Nrf2; therefore, Nrf2 can induce p62 expression during oxidative stress, and p62 can inhibit Nrf2 degradation, forming a feedforward loop between Nrf2 and p62.^117^ Protein kinases such as MAPK and PKC also contribute to Nrf2 accumulation by phosphorylating Nrf2 to dissociate Nrf2 from Keap1.^118^^,^^119^

## Regulatory Mechanisms of Nrf2 in OS

Nrf2 has been extensively investigated for more than 20 years and is considered a key regulator of the antioxidant defense system.^120^ Nrf2 protects normal cells from ROS-mediated oxidative stress capable of inducing damage to proteins, lipids, and DNA, ultimately leading to RCD. Recent studies have revealed numerous other functions of Nrf2 in cancer cells, including the regulation of drug metabolism, iron metabolism, amino acid, lipid, and glucose metabolism, DNA repair, proliferation, metastasis, and autophagy.^121^ The diversity of Nrf2-mediated functions explains its dual role in cancer progression. In normal tissues, Nrf2 maintains cellular physiological homeostasis by protecting cells from oxidative stress damage and regulating cellular detoxification, DNA repair, and immunosurveillance to protect cells from endogenous or exogenous damage, thus avoiding precancerous lesions.^122^^,^^123^ Upon tumor development, tumor cells utilize various Nrf2 functions to maintain physiological homeostasis to avoid RCD and promote malignant phenotypes, such as proliferation and metastasis, to accelerate tumor progression.^124^ In a murine model of urea-induced lung cancer, Keap1 knockout mice exhibited rapid urinary urea excretion and reduced tumor incidence, consistent with Nrf2-induced drug detoxification. However, rapid tumor progression was observed upon transplanting tumor cells from Keap1 knockout mice into wild-type mice.^125^ Elevated levels of inflammatory markers and an increased incidence of inflammation-associated colorectal cancer have also been observed in Nrf2 knockout mice.^126^ Given that Nrf2 promotes ROS scavenging and DNA damage repair, Nrf2 can antagonize radiation exposure-induced elevated ROS levels and DNA damage.^127^ Thus, Nrf2 activation can prevent tumorigenesis in both radiation- and chemically-induced skin cancers.^128^^,^^129^

In contrast to the inhibition of normal cell carcinogenesis, Nrf2 can enhance cancer cell proliferation and metastasis or inhibit various stress responses, thus maintaining the physiological homeostasis of cancer cells.^130^ This protective effect promotes the immune escape of cancer cells from RCD and resistance to chemoradiotherapy. Nrf2 expression was substantially higher in human and canine OS tissues than that in normal paracancerous tissues. Furthermore, Keap1 expression was markedly lower in human and canine OS tissues than that in paracancerous tissues.^131^ Positive Nrf2 expression negatively correlated with the five-year survival of patients with OS, in contrast to the positive expression of Keap1, which was positively correlated with five-year survival.^132^ DDP-resistant OS cells exhibit higher expression of Nrf2 and GPX4 than chemotherapy-sensitive OS cells, and downregulated Nrf2 and GPX4 expression could markedly reverse OS resistance.^133^ Nrf2 plays a key regulatory role in several pathophysiological processes, including drug metabolism, RCD, and DNA repair in OS cells, and promotes OS cell proliferation and metastasis.

### Nrf2 promotes chemoradiotherapy resistance in OS

**Figure 3:**
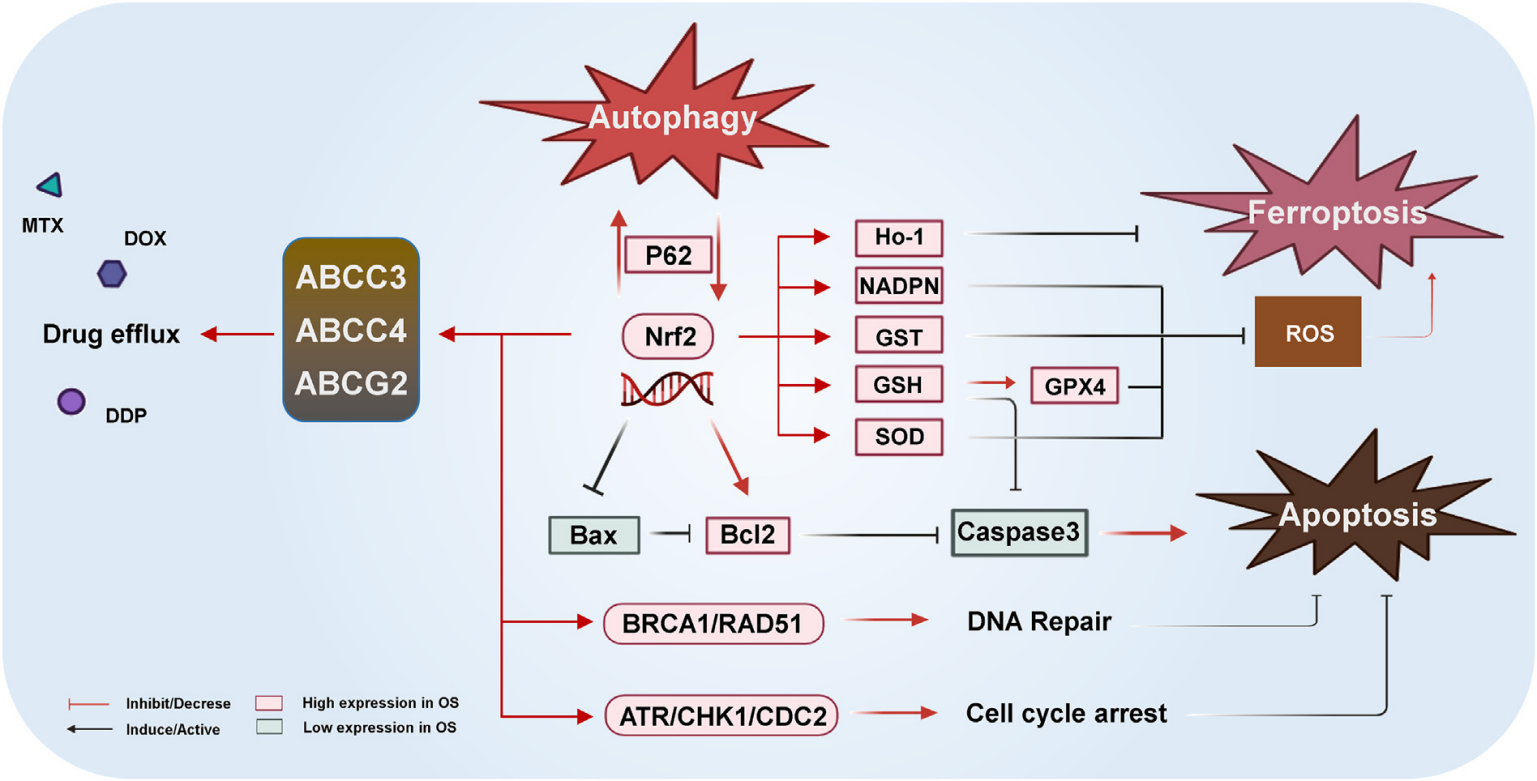
Mechanisms of Nrf2-mediated resistance to chemoradiotherapy in osteosarcoma. Hyperactivation of Nrf2 and elevated expression of its downstream genes mediate chemoradiotherapy resistance in osteosarcoma by promoting drug efflux, blocking RCD, promoting DNA repair and cell cycle arrest. RCD, regulated cell death.

As mentioned earlier, cancer cells are exposed to high levels of oxidative stress owing to their active metabolism, and multiple chemotherapeutic drugs and radiotherapy can induce antitumor effects by increasing ROS levels in cancer cells. Nrf2, a key antioxidant factor, is crucial for alleviating oxidative stress in OS cells and promoting resistance to chemoradiotherapy.^134^^,^^135^ Nrf2 expression was substantially elevated in OS-resistant cells, as well as in Nrf2-mediated OS MDR, by increasing the antioxidant activity of OS cells (Fig. 3). DDRGK domain-containing protein 1 (DDRGK1) binds to Keap1 competitively to promote Nrf2 degradation, and DDRGK1 knockdown in OS cells could promote ROS accumulation and enhance OS cell sensitivity to DOX and etoposide.^136^^,^^137^ Elevated ROS levels can mediate apoptosis or ferroptosis in OS cells, and inhibition of Nrf2 expression in OS cells can trigger apoptosis by increasing ROS levels.^138^ Mechanistically, Nrf2 exerts its antioxidant effects by regulating the expression of various antioxidant genes containing ARE sequences, including GSH, GST, NADPH, superoxide dismutase (SOD), and heme oxygenase (HO)-1.^139^ ATO-treated OS cells reportedly display Nrf2 nuclear translocation and increased HO-1, GSH, and SOD expression. GSH and SOD exert antioxidant effects and inhibit caspase-3 activity and apoptosis, whereas HO-1 failed to elicit a notable effect.^140^^,^^141^ However, the Nrf2/HO-1/GPX4 axis was found to be crucial for reducing ROS levels and preventing ferroptosis in cancer cells and is an important target for chemotherapy drugs.^142^ Nrf2 prevents apoptosis by upregulating the antiapoptotic protein Bcl-2, downregulating the apoptotic protein Bax, and regulating the expression of antioxidant factors.^143^^,^^144^ Thus, the antiapoptotic function of Nrf2 is achieved via multiple mechanisms, exerting a tumor-preventive effect in normal cells and cellular resistance in OS cells.^145^^,^^146^ Nrf2 reportedly modulates various drug transporter proteins, decreasing the concentration of chemotherapy drugs in tumor cells and achieving drug resistance through increased drug efflux.^147^ In OS cells, siRNA-mediated downregulated Nrf2 expression downregulated the expression of the ABC family (ABCC3, ABCC4, and ABCG2) and reversed OS cell resistance to DDP, DOX, and sorafenib.^148^

The use of radiotherapy in OS remains controversial owing to its poor efficacy and tolerability.^149^ Patients with OS receiving chemoradiotherapy have similar 5-year survival rates to those receiving chemotherapy, although the incidence of traumatic complications was higher.^150^ The antitumor effect of radiotherapy is primarily achieved via the induction of increased ROS levels and DNA breakage, and the antioxidant function of Nrf2 is critical for radiotherapy-mediated OS cell resistance. Therefore, targeting Nrf2 as an adjuvant to radiotherapy for OS could be a promising approach. Animal experiments have demonstrated that suppressing Nrf2 levels could increase ROS levels in cancer cells, inhibiting DNA repair and thereby increasing sensitivity to radiotherapy.^151^ In OS cells, siRNA-mediated inhibition of Nrf2 expression and irradiation revealed that elevated ROS levels could induce mitochondrial membrane damage, which, in turn, generated additional ROS. Nrf2 inhibition prolonged the DSB repair time, substantially reducing the DNA repair rate when compared with that in the control group.^152^ Considering ROS elimination, DSBs can induce Nrf2 expression and recruit Nrf2 to DNA-damaged sites, leading to G2 cell cycle arrest and promotion of DSB repair.^153^ Mechanistically, Nrf2 improves homologous recombination (HR) efficiency by increasing the expression of BRCA1 and RAD51, two essential regulatory proteins for HR. Nrf2 interacts with ATR to activate the ATR/CHK1/CDC2 signaling pathway, leading to cell cycle arrest.^154^ Similar to Nrf2, autophagy reduces cellular oxidative stress and cytotoxicity by scavenging excess ROS and transporting damaged DNA outside the nucleus, thereby mediating chemotherapy resistance in OS cells.^155^ Interestingly, P62, a key protein in autophagy, can promote the nuclear translocation of Nrf2 by competitively binding to Keap1, which, in turn, promotes P62 expression in a feedforward manner.^156^ This facilitates the rapid removal of damaged DNA to reduce cytotoxicity during early-stage DNA damage, thereby preventing excessive DNA damage-mediated autophagic RCD.^157^ Autophagy, as a regulatory center for cellular nutrient metabolic activity, promotes the clearance and reuse of macromolecules in tumor cells. Reportedly, autophagy can also contribute to extracellular nutrient acquisition by tumor cells through macropinocytosis, another mode of nutrient acquisition, which is dependent on P62-mediated Nrf2 activation, suggesting that the combined application of autophagy and Nrf2 inhibitors has clinical therapeutic potential for cancer.^158^^,^^159^ In radiotherapy-irradiated OS cells, autophagy increased Nrf2 expression by promoting ERK1/2 phosphorylation, resulting in radiotherapy resistance; however, the knockdown of autophagy proteins reversed the OS cell resistance.^160^ Collectively, these findings suggest the existence of a crosstalk between autophagy and Nrf2, and elucidating the underlying mechanisms is crucial for developing antitumor drugs.

### Nrf2 promotes progression of OS

With accumulating evidence on Nrf2, numerous other Nrf2 functions have been discovered, including the promotion of cancer cell proliferation and metastasis. Nrf2 overexpressing mice showed robust cellular antioxidant stress and cell proliferation. Conversely, Nrf2 knockout mice showed slower cell proliferation than wild-type mice.^161^^,^^162^ Sequencing of Keap1 and Nrf2 knockout mouse embryonic fibroblasts revealed that Nrf2 can regulate the expression of proliferation- and metastasis-related genes.^163^ In OS cells, Keap1 overexpression markedly suppressed proliferation, which was reversed by Nrf2 inhibition.^164^ MAFG can form a heterodimer with Nrf2 to bind downstream ARE sequences, and MAFG knockdown in OS cells substantially reduced the *in situ* growth of mouse tibial graft tumors.^165^ To maintain proliferative demands, malignant tumors metabolize faster than normal cells and have an increased glucose demand, rendering glycolysis a major energy source for cancer cells.^166^ Moreover, Nrf2 was found to be a key regulator of metabolic processes in tumor cells, exerting regulatory effects that influence cancer cell proliferation and intracellular redox homeostasis. Nrf2 regulates NADPH production by modulating key enzymes of the pentose phosphate pathway, including glucose 6-phosphate dehydrogenase, 6-glucose phosphate dehydrogenase, and isocitrate dehydrogenase 1.^167^ NADPH is an important reductive substance in the body and acts as a cofactor in diverse antioxidant pathways, which is another role of Nrf2 in exerting antioxidant functions by regulating nutrient metabolism. Nrf2 reportedly increases the expression of key enzymes in glucose metabolism and enhances the glucose uptake rate to provide nutrients for cancer cell proliferation.^168^

OS cell metastasis depends on the co-regulation of multiple mechanisms, including epithelial–mesenchymal transition (EMT), angiogenesis, and blockade of apoptosis. In OS cells, suppression of Nrf2 expression substantially inhibits cell proliferation and migration, in which Nrf2 plays a complex role.^165^ Notably, cancer cell metastasis must return to the epithelial phenotype to lose contact with neighboring cells and enter the metastasized area before proliferation continues, a process known as EMT, regulated by multiple regulatory proteins.^169^ In OS cells, downregulated Nrf2 expression resulted in the inhibition of EMT. Several EMT-related proteins have been identified, including upregulated E-cadherin and downregulated N-cadherin and vimentin.^170^ Nrf2 can reportedly mediate cancer cell migration by downregulating Notch1, an important regulator of EMT.^171^ EMT in cancer cells is also driven by hypoxia, with a recent study reporting that hypoxia drives EMT by increasing Nrf2 expression via HIF-1α. Under hypoxic conditions, a bystin-like gene was found to drive EMT in OS cells in an Nrf2-dependent manner.^172^ Y-box binding protein 1 (YB-1) contributed to various cancer malignant phenotypes, including DNA repair, cell proliferation and differentiation, and migration. Reportedly, Nrf2 may be a novel target of YB-1, mediating migration in OS and Ewing sarcoma.^173^

Collectively, Nrf2 mediates chemoradiotherapy resistance and OS progression by modulating multiple cytokines. Based on accumulated evidence, Nrf2 can exert diverse regulatory functions in addition to antioxidant stress, and crosstalk exists between Nrf2 and autophagy; hence, elucidating these mechanisms would be highly valuable.

## Nrf2-based clinical trials

Nrf2 is an important transcription factor regulating the dynamic balance of cellular redox reactions. Moreover, Nrf2 plays a key role in tumor resistance to chemoradiotherapy, proliferation, and migration; therefore, it is a markedly promising antitumor strategy to promote ROS production and suppress key signaling pathways, such as proliferation, migration, and DNA repair by inhibiting the Keap1/Nrf2/ARE pathway. Identifying effective Nrf2 inhibitors to enhance sensitivity toward chemoradiotherapy and inhibit tumor progression is a hot and difficult topic in clinical research (Table 1). High-throughput screening of small-molecule inhibitors has dramatically accelerated modern drug discovery and development. The probe molecule ML385, screened from among 400,000 different small molecules, binds to Nrf2 and interferes with Nrf2-MAFG binding, thereby inhibiting downstream molecular activation. Co-administration of ML385 with DOX or paclitaxel elicited robust antitumor effects in preclinical lung cancer models. Notably, ML385 exhibited high specificity for Keap1-mutated cells.^174^ In OS mice, miR-4660-mediated MAGF inhibition suppressed the *in situ* growth of OS cells in the proximal tibia.^165^ APE1 is a multifunctional protein, and early studies have found that APE1 is crucial for Nrf2 activation. Interference with APE1 substantially enhanced oxidative stress *in vivo* and increased chemoradiotherapy resistance in a mouse-transplanted lung cancer model.^175^ In addition to antioxidant stress, APE1 was recently shown to regulate the DNA damage response through ARE-Chk1 signaling, which can be suppressed by siRNA intervention, corresponding to the DNA repair-promoting function of Nrf2.^176^ Currently, the main approaches for regulating Nrf2 expression include epigenetic regulation and protein interactions, in addition to the use of active ingredients from Chinese herbs, which afford new directions for drug development. For example, oridonin, an active ingredient extracted from *Rabdosia rubescens*, can exert favorable anticancer effects. In OS model mice, oridonin increased the Bax/Bcl-2 ratio and cleavage of Caspase-3 and Caspase-9 dose-dependently, in addition to increasing PPAR-γ expression and suppressing the nuclear translocation of NF-κB and Nrf2 in OS tissues. Oridonin-treated OS mice showed a marked reduction in tumor volume and enhanced apoptosis, although there was no significant change in body weight or major organs during the 21-day treatment period. Accordingly, oridonin could be a safe and effective anti-OS drug.^177^ Previously, we have shown that *Oculina diffusa* combined with DDP exerted promising antitumor effects, and the extracted active ingredient, quercetin, could upregulate proapoptotic proteins and downregulate antiapoptotic proteins in OS cells.^178^^,^^179^ Interestingly, our recent experimental data also suggested that Nrf2 is an important target of quercetin.

**Table 1:** Nrf2-based preclinical studies and clinical trials.

Interventions	Target	Administration	Model Type	Results	Ref
Preclinical Studies					
ML385	Neh1	intraperitoneal injection	NSCLC	Enhanced antitumor and anti-metastatic effects when combined with DDP therapy	174
Mir-4660	MAFG	Intratumoral injection	Osteosarcoma	Decreased tumor volume	165
APE1 knockout tumors	Nrf2	intraperitoneal injection	NSCLC	Reduction of antioxidant stress capacity	175
Oridonin	PPAR-γ	intraperitoneal injection	Osteosarcoma	Induced apoptosis and decreased tumor volume	177
Clinical Trials					
Sulforaphane	Nrf2	consume	Healthy human	Useless	183
Sulforaphane	Nrf2	consume	COPD patients	Useless	184
BG-12	Nrf2	consume	Multiple sclerosis	Reduction of recurrence rate	185

COPD, chronic obstructive pulmonary disease; DDP, cisplatin; Nrf2, Nuclear factor E2-related factor 2; NSCLC, Non-small cell lung cancer.

Although targeting Nrf2 has achieved favorable efficacy in various animal models, progress in clinical trials remains limited. Completed Nrf2-based clinical trials have involved the activation of Nrf2 expression using the broccoli sprout extract sulforaphane (SFN) as the interventional agent, which could be safer, as Nrf2 acts as a key regulator of antioxidant activity and radical regulation of Nrf2 expression could damage normal tissues or organs and produce serious side effects, such as myocardial or brain damage.^180^^,^^181^ Unfortunately, two clinical studies revealed that SFN intervention failed to substantially alter the expression of Nrf2 and its downstream antioxidant genes. In a study that provided healthy volunteers with 200 g of broccoli homogenate per day for three days, antioxidant gene expression remained unaltered, while plasma SFN levels were substantially elevated (NCT01625130). *In vitro*, SFN enhanced cellular antioxidant function by activating Nrf2 expression. However, the increased SFN levels were markedly lower than those observed *in vitro*, and this “mild” supplementation therapy was probably insufficient to activate Nrf2 through SFN.^182^^,^^183^ In patients with COPD administered 25 or 150 μmoL oral SFN daily for four weeks (NCT01335971), both doses failed to stimulate the expression of Nrf2 or antioxidant genes despite the increased exposure time and dose.

Notably, BG-12 (dimethyl fumarate) was the first Nrf2 activator with satisfactory efficacy approved by the US Food and Drug Administration to treat multiple cerebrospinal sclerosis.^184^ However, the clinical application of Nrf2 inhibitors for antitumor therapy presents considerable challenges, given that the harmful effects of Nrf2 deficiency on normal cells can be fatal. The safe and effective antitumor effects of Nrf2 inhibitors depend on precise and efficient drug delivery systems. For example, inhalational delivery of nanoparticle-packaged drugs to target lung lesions could effectively reduce systemic toxicities and was found to be safe and efficient.^185^ Furthermore, we have recently reported that quercetin encapsulated in 190 nm liposomes modified with folate could exert robust drug targeting with limited off-target effects. Importantly, the antitumor effects of folate-modified drugs are substantially enhanced owing to the high overexpression of folate receptors on the OS cell surface.^186^

## Discussion

Based on the growing research on Nrf2 and the gradual discovery of new target genes and their functions, Nrf2 can act as an oncogene that promotes tumorigenesis and progression through various mechanisms. In OS, Nrf2 hyperactivation mediates resistance to chemoradiotherapy through mechanisms such as antioxidant stress, promotion of DNA repair, reduction in drug concentration, and promotion of cell proliferation and migration to accelerate OS progression. Although some controversy remains, functions such as the regulation of cell proliferation and metastasis in OS reflect the oncogenic signature of Nrf2. Therefore, Nrf2, as an important regulator of multiple functions, can be deemed a promising therapeutic target, either as an adjunct to chemoradiotherapy to enhance sensitivity or as a novel immunotherapy.

Since the discovery of the tumor-promoting effects of Nrf2, the development of effective and safe Nrf2 inhibitors has gained considerable momentum; however, the results remain limited. Dimethyl fumarate is the only Nrf2 activator approved by the FDA to treat multiple cerebrospinal sclerosis.^184^ The development of Nrf2 inhibitors faces additional challenges and limitations owing to the dual role of Nrf2, including myocardial and cerebral toxicity owing to downregulated Nrf2 expression.^180^^,^^181^ Currently, there are two main strategies for Nrf2 regulation: epigenetic regulation and protein interactions; the former mainly regulates Nrf2 through siRNAs and noncoding RNAs, while the latter involves diverse Nrf2 inhibitors and active ingredients isolated from Chinese herbs. Previously, we have identified the anti-OS function of quercetin mediated by promoting oxidative stress and apoptosis, and our recent data suggests that Nrf2 may be an important target.^178^^,^^179^ Quercetin encapsulation in folate-modified liposomes elicited robust targeting properties and efficacy owing to the high expression of folate receptors on the OS cell surface.^186^ Recently, various miRNAs have been found to exert antitumor effects by regulating Nrf2 expression in OS. Drug delivery systems for miRNAs include liposomes, nanoparticles, and exosomes. These delivery systems possess inherent advantages and disadvantages; therefore, selecting an optimal delivery system is crucial for the clinical application of Nrf2 inhibitors.

Furthermore, the application of Nrf2 inhibitors depends on the type and stage of cancer and the patient's overall status, and understanding how to balance the cancer-preventive and -promoting functions of Nrf2 is necessary to provide clinical benefits to patients. Therefore, the dual role of Nrf2 needs to be comprehensively clarified, which remains a necessary and difficult task. We have recently reviewed the advantages and disadvantages of different animal models for OS studies, and desirable animal models are valuable for revealing the critical mechanisms of Nrf2.^187^ For example, organoid models exhibit stable phenotypic and genetic profiles and can be obtained from patient-derived healthy or tumor tissues in culture. A normal and drug-resistant OS culture system based on an organoid model, as well as an exploration of key mechanisms through which Nrf2 promotes OS resistance and progression, would better reflect the real scenario in the organism.

## Funding

This work was supported by The National Natural Science Foundation of China (No. 82274559).

## Conflict of interests

The authors declare that they have no conflicts of interest.
